# Sulbactam for carbapenem-resistant *Acinetobacter baumannii* infections: a literature review

**DOI:** 10.1093/jacamr/dlaf055

**Published:** 2025-04-12

**Authors:** Nikolaos Spernovasilis, Angela Ishak, Constantinos Tsioutis, Danny Alon-Ellenbogen, Aris P Agouridis, Nikolaos Mazonakis

**Affiliations:** Department of Infectious Diseases, German Medical Institute, 4108 Limassol, Cyprus; Department of Internal Medicine, Henry Ford Hospital, 48202 Detroit, MI, USA; School of Medicine, European University Cyprus, 2404 Nicosia, Cyprus; Department of Basic and Clinical Sciences, University of Nicosia, 2417 Nicosia, Cyprus; School of Medicine, European University Cyprus, 2404 Nicosia, Cyprus; Department of Internal Medicine, German Medical Institute, 4108 Limassol, Cyprus; Department of Internal Medicine, Thoracic Diseases General Hospital Sotiria, 11527 Athens, Greece

## Abstract

Carbapenem-resistant *Acinetobacter baumannii* (CRAB) is characterized as a critical priority pathogen with restricted therapeutic options. To date, the most effective antimicrobial treatment against this difficult-to-treat bacterial strain has not been established. Sulbactam is a β-lactamase inhibitor with intrinsic activity against this pathogen, however, as a β-lactam, it can be hydrolysed by β-lactamases produced by *A. baumannii*. High-dose, extended-infusion treatment with sulbactam can overcome this hydrolysis by β-lactamases and is considered an effective therapeutic strategy against CRAB. The aim of this review is to analyse primary and secondary research studies that compare sulbactam-based with other regimens, such as polymyxin-containing regimens, tigecycline-containing regimens and other antimicrobial combinations against CRAB infections, especially ventilator-associated pneumonia (VAP), hospital-acquired pneumonia (HAP) and bacteraemia. Our findings suggest that results are conflicting, mostly because of high heterogeneity among studies. However, in most studies, sulbactam-based regimens have demonstrated comparable, and in several studies more favourable results in contrast to other antimicrobial treatments with respect to clinical cure and mortality in CRAB-associated pneumonia, yet without reaching statistical significance in most cases. The auspicious novel β-lactam/β-lactamase inhibitor combination sulbactam/durlobactam is also discussed, although real-world clinical data regarding its efficacy in CRAB infections are still scarce. More randomized controlled trials comparing sulbactam-based with other regimens are warranted to determine the most effective antimicrobial combination against CRAB infections. Nevertheless, current data suggest that sulbactam could play a major role in this combination treatment.

## Introduction

The *Acinetobacter baumannii–calcoaceticus* complex (ABC) represents the species of the genus *Acinetobacter* with the greatest clinical relevance, causing numerous healthcare-associated infections, such as bacteraemia, respiratory tract, urinary tract and surgical wound infections.^1,2^ Carbapenem-resistant *A. baumannii* (CRAB) poses a global threat to healthcare systems due to limited therapeutic options against infections caused by this bacterial strain, leading to its characterization by the WHO as a critical priority pathogen.^3^

Treating CRAB infections entails plenty of challenges for many reasons. CRAB is often isolated from respiratory and wound samples. It is extremely difficult to distinguish a colonization from a true infection, especially in critically ill hospitalized patients with underlying co-morbidities or those immunocompromised. This can lead to significant delay in treatment initiation and substantially to poor outcomes.^4^ On the other hand, this clinical conundrum may result to overtreatment, leading to delayed ICU and hospital discharges, more adverse drug reactions and increased antimicrobial resistance.^5^ Moreover, CRAB isolates usually develop different mechanisms of resistance, such as β-lactamases, antimicrobial target modifications, increased activity of efflux pumps and alterations in the structure and number of porins, rendering them resistant to multiple categories of antibiotics.^6^ These multiple mechanisms can lead to formation of pan-drug-resistant isolates, which can cause infections whose effective management requires antimicrobial combinations aiming for a possible synergistic action.^7^ Even the use of antimicrobial agents with the highest *in vitro* activity against CRAB, such as polymyxins (colistin and polymyxin B), tetracyclines (eravacycline, minocycline and tigecycline) and ampicillin-sulbactam, requires rigorous dose-optimization strategies to achieve pharmacokinetic-pharmacodynamic (PK-PD) targets in the context of invasive CRAB infections.^8^ Finally, large, randomized studies comparing the effectiveness of the abovementioned drugs are limited and, as a result, there is no ‘gold standard’ antibiotic regimen for CRAB infections.^9^

According to the Infectious Diseases Society of America 2024 Guidance on the treatment of CRAB infections, a combination of at least two agents is suggested. It is also suggested that at least one antimicrobial in the combination is sulbactam based.^9^ The practice of using sulbactam as the backbone of the therapeutic armamentarium against CRAB isolates is still up for debate. The aim of this review is to narratively summarize evidence from clinical studies comparing sulbactam-based regimens with others regarding treatment of CRAB infections.

## Sulbactam’s activity against CRAB isolates

Sulbactam is an irreversible β-lactamase inhibitor with intrinsic activity against *A. baumannii*, saturating its PBP1a/1b and PBP3.^10^ As a β-lactamase inhibitor, this molecule exhibits variable inhibitory activity against only class A β-lactamases, whereas as a β-lactam, it can be hydrolysed by β-lactamases by all Ambler classes, including class A, *Acinetobacter* derived cephalosporinase (ADC)-type class C, OXA-type class D β-lactamases, and metallo-β-lactamases (MBL), which are commonly produced by the ABC complex.^11^ Among these β-lactamases, class D OXA-type carbapenemases are the most clinically significant, with the gene *bla_OXA-23_* being the most widespread globally, detected in up to 74.5% of *A. baumannii* isolates in Europe and 39.5% in the USA.^12^ Other OXA-type β-lactamase genes, such as *bla_OXA-24/40_* and *bla_OXA-58_*, are also prevalent but at lower frequencies.^12^ For instance, in Spain, *bla_OXA-24/40_-*like genes were identified in 48.7% of CRAB isolates in 2000 and 51.6% in 2010.^13^ Additionally, *bla_OXA-58_* has been detected in various countries, including the USA, UK, Turkey, Greece, Argentina, Kuwait, Italy, Spain and Austria, with prevalence rates varying by region.^14^ Class B metallo-β-lactamases, including the gene *bla_NDM-1_*, have been detected in *A. baumannii* isolates from Europe and the USA, although their prevalence remains relatively low (<2%).^15^ Although less common, the MBL genes *bla_IMP_* and *bla_VIM_* also contribute to broad-spectrum β-lactam resistance.^12^ Finally, class C ADC enzymes are intrinsic to *A. baumannii*, with the *bla_ADC_* gene playing a key role in cephalosporin resistance, however, mutations within this gene can broaden the hydrolytic activity of *A. baumannii* to include carbapenems.^12^

**Figure 1.**
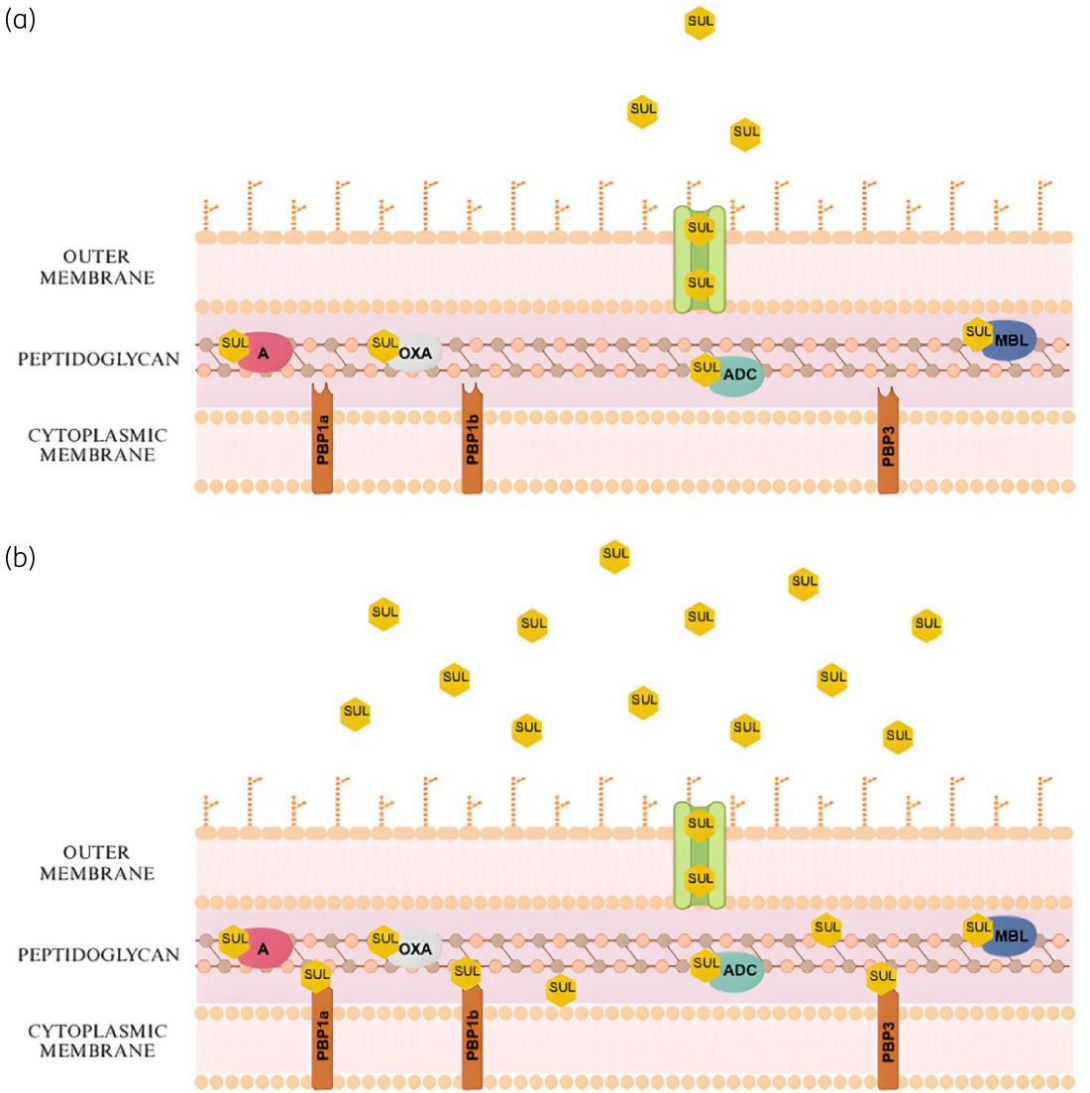
The rationale behind the use of high-dose sulbactam against CRAB infections. (a) Conventional-dose therapy with sulbactam may not be effective against CRAB, as this molecule can be hydrolysed by class A, ADC-type class C, OXA-type class D β-lactamases, and MBLs, which can be produced by CRAB isolates. As a result, sulbactam molecules cannot reach their targets, i.e. PBP1a, PBP1b and PBP3. (b) High-dose, extended-infusion therapy allows more sulbactam molecules to reach their PBP targets, as they overcome the hydrolysis by β-lactamases. Binding to PBPs leads to interruption of bacterial cell wall formation and consequently, to bacterial death. Figure created with BioRender.com.

Over the years, sulbactam has been widely commercially available in the form of ampicillin-sulbactam with a ratio of 2:1 (2 g of ampicillin, 1 g of sulbactam).^8^ As in the case of other β-lactams, the main pharmacokinetic parameter regarding its effective action, is the time that free sulbactam concentrations remain above the MIC (*f*T > MIC).^16^ However, on a global basis, most CRAB isolates test resistant to ampicillin-sulbactam.^17^ High doses of sulbactam [i.e. an intravenous (IV) 4-hour infusion of 3 g sulbactam every 8 hours] are required to achieve sufficient *f*T > MIC in case of *A. baumannii* isolates with a sulbactam MIC 16 mg/L, as demonstrated in PK studies.^16,18^ The rationale behind this therapeutic strategy is that high doses will allow more sulbactam molecules to reach their penicillin-binding protein (PBP) targets, overcoming the hydrolysis by β-lactamases (Figure 1).^9^

While high-dose sulbactam is necessary to achieve therapeutic efficacy against *A. baumannii*, critically ill patients and those with renal impairment may require augmented dosing to optimize drug exposure to minimize drug toxicity. However, the safe and optimal dosing of sulbactam/ampicillin in these populations remains poorly defined. Early pharmacokinetic studies demonstrated that renal impairment significantly prolongs sulbactam elimination, necessitating dose adjustments to prevent toxicity.^19,20^ Blum *et al.* found that in severe renal failure, the half-life of sulbactam and ampicillin more than doubled, while haemodialysis enhanced drug clearance, underscoring the need for modified dosing.^19^ Lorenzen *et al.* demonstrated that in critically ill patients with acute kidney injury (AKI) on extended dialysis, standard sulbactam dosing resulted in underdosing, necessitating a twice-daily 2 g/1 g regimen to maintain therapeutic levels.^21^ Additionally, Yokoyama *et al.* found that sulbactam clearance was significantly associated with renal function, indicating the necessity for dosage adjustments based on creatinine clearance to maintain therapeutic efficacy while minimizing toxicity.^22^ Despite these findings, robust pharmacokinetic data guiding safe sulbactam dosing in renally impaired patients remain limited, which presents a challenge for the routine use in this population.

Beyond dosing optimization, sulbactam’s activity can be enhanced by combining it with β-lactamase inhibitors or other β-lactams to overcome resistance. For instance, lower doses of sulbactam can be used when combined with durlobactam, a potent inhibitor of class A, C and D β-lactamases produced by CRAB isolates, enabling sulbactam molecules to saturate PBPs before their inactivation.^23^ Moreover, *in vitro* studies have demonstrated that other β-lactamase inhibitors, such as avibactam and relebactam, can also reduce the MIC of sulbactam against multidrug-resistant *A. baumannii* isolates.^24,25^

Moreover, given sulbactam’s ability to achieve high epithelial lining fluid (ELF) concentrations, sulbactam presents a promising therapeutic option for pulmonary infections caused by CRAB. This was shown by a recent PK/PD analysis in the neutropenic murine pneumonia model, a 4-hour infusion of 3 g sulbactam every 8 hours achieved adequate ELF exposures (probability of target attainment >90%) for *A. baumannii* isolates with sulbactam MIC up to 8 mg/L.^26^ On the other hand, the insufficient penetration of IV colistin into pulmonary ELF raises concerns regarding its efficacy against *A. baumannii* pneumonia.^27^ Nevertheless, it is of the utmost importance to examine how these data are reflected in clinical studies.

## Sulbactam-based versus other regimens against CRAB infections

Clinical safety of high-dose ampicillin-sulbactam in case of MDR-*A. baumannii* infections has been demonstrated formerly.^28^ In a randomized controlled trial conducted in Greece, patients were randomly assigned to receive either 9 g (*n* = 14) or 12 g (*n* = 13) of sulbactam per day for the treatment of MDR-*A. baumannii* ventilator-associated pneumonia (VAP). According to the results, no major adverse events were reported.^28^ However, clinical data are scarce with reference to direct comparison between sulbactam-based and other regimens in the context of CRAB infections. In this section studies comparing sulbactam-based with other therapeutic regimens in adult patients with CRAB infections are analysed. Primary clinical studies on sulbactam-based versus other treatments are also summarized in Table 1.

**Table 1.** Primary research studies comparing sulbactam-based with other regimens in the treatment of CRAB-associated infections

Author (year)	Type of study	Location	Type of infection	Number of patients	Therapeutic regimens	Total daily dose of sulbactam	Sulbactam MIC	Major findings
Batirel *et al.* (2014)^29^	Retrospective study	Turkey (multicentre)	XDR-*A. baumannii* bloodstream infection	250	CST monotherapy (*n* = 36), CST plus Carbapenem (*n* = 102), CST plus SUL (*n* = 69), CST plus TGC or an aminoglycoside or RIF or TZP (*n* = 43)	4–6 g of SUL/day	N/A	Significantly higher microbiological eradication rate (79.9% versus 55.6%, *P *= 0.001), and significantly lower in-hospital mortality rate (52.3% versus 72.2%, *P *= 0.03), in favour of the combination treatment group. No significant differences regarding clinical and microbiological response, as well as 14-day survival rates among the three combination groups.
Betrosian *et al.* (2008)^30^	Randomized clinical trial	Greece (two-centre)	CRAB-VAP	28	HD SAM (*n* = 13) versus CST (*n* = 15)	9 g of SUL/day	SAM MIC > 32/16 mg/L (SUL MIC > 16 mg/L)	No statistically significant difference in clinical success, bacteriological success, 14-day mortality and 28-day mortality rates.
Kalin *et al.* (2014)^31^	Retrospective study	Turkey (single centre)	MDR-*A. baumannii* VAP (>90% of isolates carbapenem-resistant)	89	CST plus HD SUL (CFP/SUL) (*n* = 37) versus CST monotherapy (*n* = 52)	9 g of SUL/day	N/A (77% reported resistance rate to CFP/SUL)	No statistically significant difference in early response, clinical response, bacteriological response and mortality rates.
Kaye *et al.* (2023)^32^	Phase 3, multinational, randomized, active-controlled, non-inferiority trial	16 countries (multicentre)	CRAB infection (VAP, HAP and bacteraemia)	125	CST (*n* = 62) versus SUL/DUR (*n* = 63) combined with IPM as background therapy	4 g of SUL/day	91% of isolates with a SUL MIC > 8 mg/L (Resistance), 5% of isolates with a SUL MIC = 8 mg/L (Intermediate susceptibility), 5% of isolates with a SUL/DUR MIC > 4 mg/L (Resistance)	Lower 28-day all-cause mortality in the SUL/DUR group (19.0% versus 32.3%) (non-inferior). Significantly higher clinical cure rates in the SUL/DUR group (62% versus 40%).
Khalili *et al.* (2018)^33^	Open-label, randomized clinical trial	Iran (single centre)	CRAB-VAP	47	MEM plus HD SAM (*n* = 23) versus MEM plus CST (*n* = 24)	6 g of SUL/day	N/A	No statistically significant differences in clinical response, microbiological response and 28-day mortality rates.
Khawcharoenporn *et al.* (2014)^34^	Retrospective study	Thailand (single centre)	XDR-*A. baumannii* pneumonia	166	neb or IV CST plus HD SUL (*n* = 93), neb or IV CST plus TGC (*N* = 43), neb or IV CST plus Carbapenem (*n* = 30)	6 g of SUL/day	N/A (all isolates were reported as resistant to SUL)	No statistically significant differences regarding 28-day survival rates between the 3 subgroups of patients.
Lee *et al.* (2013)^35^	Retrospective study	Taiwan (single centre)	MDR-*A. baumannii* infection (VAP, HAP, bacteraemia and other infections)	386	SUL plus IPM (*n* = 120) versus TGC alone or combined with a carbapenem or TZP or a 3rd generation cephalosporin (*n* = 266)	4 g of SUL/day	N/A (all isolates were reported as resistant to SUL)	No significant differences in 30-day mortality and length of hospital and ICU stay.
Liang *et al.* (2018)^36^	Retrospective study	Taiwan (multicentre)	CRAB pneumonia (VAP: 58.8%)	238	TGC monotherapy (*n* = 84), TGC plus CST (*n* = 43), CST monotherapy (*n* = 34), CST plus other antimicrobials (*n* = 33), TGC plus other antimicrobials (*n* = 32), SUL-based therapy (*n* = 12)	4 g of SUL/day	N/A (19.5% of isolates were reported as susceptible to SAM)	TGC monotherapy was associated with the highest crude ICU mortality (37/84, 44.0%, *P *= 0.031), whereas SUL-based therapy with the lowest (1/12, 8.3%). In multivariate analysis, tigecycline-based therapy was associated with higher ICU mortality than non-tigecycline therapy (adjusted OR 2.30, 95% CI 1.19–4.46). No difference between colistin-based therapy and non-colistin therapy regarding ICU mortality.
Lv *et al.* (2020)^37^	Randomized clinical trial	China (single centre)	MDR-*A. baumannii* pneumonia	114	CFP/SUL plus TGC (*n* = 57) versus CFP/SUL monotherapy (*n* = 57)	3 g of CFP/SUL every 8 hours	N/A	Significantly lower serum levels of PCT, CRP, TNF-a and IL-6 (all *P *< 0.001), as well as significantly lower APACHE II score (*P *< 0.001) in favour of the combination treatment group, after 14 days of treatment.
Makris *et al.* (2018)^38^	Prospective, open-label, randomized study	Greece (two-centre)	CRAB-VAP	39	CST plus HD SAM (*n* = 20) versus CST monotherapy (*n* = 19)	8 g of SUL/day	SAM MIC < 16 mg/L (at least intermediate susceptibility to SAM)	Higher early clinical response rates in favour of combination therapy (70% versus 15.8%, *P *= 0.001). No statistically significant difference in 28-day mortality rates. Association of initial favourable response with survival and discharge from ICU in multiple regression analysis.
Mosaed *et al.* (2018)^39^	Single-blind, randomized clinical trial	Iran (single centre)	CRAB-VAP	23	HD SAM plus IV LVX (*n* = 12) versus CST plus IV LVX (*n* = 11)	8 g of SUL/day	N/A	Significantly higher clinical response rate (83.3% versus 27.3%, *P *= 0.007), lower 14-day mortality rate (8.3% versus 72.7%, *P *= 0.002), lower 28-day mortality rate (41.7% versus 81.8%, *P *= 0.04) and lower incidence of AKI (8.3% versus 54.5%, *P *= 0.016) in favour of the SAM-treated group.
Niu *et al.* (2019)^40^	Retrospective study	China (single centre)	CRAB-bloodstream infection	210	TGC (*n* = 135) versus CFP/SUL (*n* = 75) (both in combination treatment with other antimicrobials)	1–2 g of CFP/SUL every 6 or 8 hours	>90% of isolates were reported as resistant to SAM (MIC >16/8 mg/L) [CFP/SUL susceptibility was based on the breakpoints for SAM (MIC of 16/8 mg/L)]	Significantly lower 28-day mortality among CFP/SUL-treated patients (29.3% versus 51.9%, *P *= 0.001).
Oliveira *et al.* (2008)^41^	Retrospective study	Brazil (two-centre)	CRAB infection (bloodstream infection: 47%, pneumonia: 32%, surgical site infection: 17% and other infections: 4%)	167	Polymyxin B or CST (*n* = 82) versus SAM (*n* = 85) (in combination treatment with other antimicrobials, such as VAN, carbapenems and aminoglycosides)	maximum daily dose of SUL: 4 g	N/A	Higher clinical cure and improvement rates, as well as lower mortality rates in the SAM-treated group of patients. Treatment with polymyxins was independently associated with death during treatment when compared with treatment with SAM.
Pourheidar *et al.* (2019)^42^	Open-label, randomized clinical trial	Iran (single centre)	CRAB-VAP	28	HD SAM plus neb CST (*n* = 12) versus IV CST plus neb CST (*n* = 16)	8 g of SUL/day	N/A (75% reported resistance rate to SAM)	No statistically significant differences regarding microbiological eradication, clinical improvement, survival rate, length of hospital and ICU stay. Lower nephrotoxicity rate in the SAM-treated group of patients.
Qin *et al.* (2018)^43^	Randomized clinical trial	China (single centre)	XDR-*A. baumannii* VAP	42	CFP/SUL plus TGC (*n* = 21) versus TGC monotherapy (*n* = 21)	3 g of CFP/SUL every 6 hours	MIC range for CFP/SUL: 64/64–1024/1024 mg/L	Significantly higher total combined effectiveness rate in favour of the combination treatment group (85.7% versus 47.6%, *P *= 0.010).
Seok *et al.* (2021)^44^	Retrospective study	Republic of Korea (multicentre)	CRAB infection (pneumonia: 91.1%, primary bacteraemia: 7.1%, UTI:1.4%)	282	CST-based, Carbapenem-based, SUL-based, TGC-based, MIN-based and AMK-based regimens	N/A	N/A (<10% of isolates were reported as susceptible to SUL)	In multivariate analysis: colistin plus carbapenem significantly reduced 7-day mortality, sulbactam-containing regimen significantly decreased 28-day mortality, whereas colistin monotherapy significantly increased 28-day mortality. In the subgroup analysis: sulbactam-containing regimen significantly reduced 28-day mortality in CRAB pneumonia.
Ungthammakhun et al. (2019)^48^	Prospective cohort study	Thailand (single centre)	XDR-*A. baumannii* VAP/HAP	182	CST plus SUL (*n* = 92) versus CST plus IPM or MEM (*n* = 90)	maximum dose of SUL: 6 g/day	N/A (all isolates were reported as resistant to SUL)	No statistically significant differences in 7-day, 14-day and 28-day mortality rates.
Ye *et al.* (2016)^45^	Retrospective study	Taiwan (single centre)	MDR-*A. baumannii* pneumonia	168	84 TGC-treated patients matched to 84 SUL-treated patients (combination treatment with other antimicrobials, such as MEM, IPM, CST, LVX and cephalosporins)	3–4 g of SUL/day	N/A	Significantly higher microbiological eradication rate in favour of the SUL-treated group (63.5% versus 33.3%, *P *< 0.0001). No significant differences in terms of clinical resolution, 30-day mortality and mortality during treatment.
Yilmaz *et al.* (2015)^46^	Retrospective study	Turkey (single centre)	CRAB-VAP	70	CST plus SUL (*n* = 20), CST plus IPM or MEM (*n* = 33), CST monotherapy (*n* = 17)	3 g of SUL/day	N/A (only 2 isolates were reported as susceptible to sulbactam)	No statistically significant differences in clinical response, microbiological response and 28-day mortality rates.
Zalts *et al.* (2016)^47^	Retrospective study	Israel (single centre)	CRAB-VAP	98	SAM (*n* = 32) versus CST (*n* = 66)	4 g of SUL/day	N/A (SAM was administered against SAM-susceptible isolates)	Decreased 30-day mortality (9.4% versus 25.8%, *P = *0.07), lower microbiological failure rate at day 7 (18% versus 48%, *P = *0.03), and less significant elevation in creatinine levels in the SAM subgroup of patients. Similar clinical cure rates between the two groups (56% versus 47%, *P = *0.39).

HD, high-dose; SAM, ampicillin-sulbactam; SUL, sulbactam; CST, colistin; IPM, imipenem/cilastatin; MEM, meropenem; LVX, levofloxacin; TGC, tigecycline; CFP/SUL, cefoperazone-sulbactam; RIF, rifampicin; TZP, piperacillin-tazobactam; VAN, vancomycin; MIN, minocycline; AMK, amikacin; SUL/DUR, sulbactam–durlobactam; neb, nebulized; PCT, procalcitonin; N/A, not available.

### Sulbactam-based regimens versus other treatments against CRAB pneumonia

#### Sulbactam-polymyxins

A series of studies have been conducted, comparing the efficacy of sulbactam- and polymyxin-based regimens in various combinations against CRAB respiratory infections, especially VAP. A randomized controlled study performed at two ICUs in Greece, compared directly the efficacy and safety of high-dose ampicillin-sulbactam versus colistin as monotherapies for the treatment of MDR-*A. baumannii* VAP. A total of 28 patients were included and randomly assigned to receive either 3 MIU of colistin every 8 hours (*n* = 15) or 9 grams of ampicillin-sulbactam every 8 hours (*n* = 13). All isolates were resistant to sulbactam with an MIC > 16 mg/L. As regards clinical success, mortality and adverse event rates, there were no statistically significant differences between the two study arms, although fewer cases of nephrotoxicity were observed in the ampicillin-sulbactam subgroup of patients.^30^ In a more recent retrospective study conducted in a single centre in Israel, ampicillin-sulbactam exhibited more favourable results.^47^ Researchers evaluated 98 patients with CRAB-VAP who were treated with either colistin (*n* = 66) or ampicillin-sulbactam (*n* = 32). According to their findings, treatment with colistin was associated with higher 30-day mortality (25.8% versus 9.4%, *P = *0.07), higher microbiological failure rates at 7 days of treatment (48% versus 18%, *P = *0.03) and more significant elevation in creatinine levels. Clinical cure rates at 7 days were comparable between the two groups of patients (47% versus 56%, *P = *0.39).^47^

Another randomized study was carried out at two ICUs in Greece, including 39 patients with MDR-*A. baumannii* VAP, who received either colistin monotherapy or combination treatment with high-dose ampicillin-sulbactam (8 g of sulbactam per day). All isolates were susceptible to colistin and demonstrated at least intermediate susceptibility to ampicillin-sulbactam (MIC for ampicillin-sulbactam <16 mg/L). Early clinical response rates were significantly higher in the combination treatment group of patients (70% versus 15.8% in the monotherapy group *P *= 0.001) and in multiple regression analysis, initial favourable response was associated with survival and discharge from ICU.^38^ These results are not compatible with the findings of a former retrospective study of 89 patients with MDR-*A. baumannii* VAP (>90% resistance to carbapenems) in Turkey. Researchers compared clinical cure and microbiological response rates between colistin monotherapy and colistin/sulbactam combination therapy, using 9 g of sulbactam daily. According to the results, at the end of treatment course they did not demonstrate significantly higher clinical response and microbiological eradication rates in favour of combination therapy. Additionally, when the mortality rates were adjusted for APACHE II score, no statistically significant difference was observed.^31^ Moreover, an observational study from Turkey of 70 VAP patients due to MDR or XDR-*A. baumannii*, did not reveal significant differences regarding clinical and microbiological efficacy, as well as mortality, among patients who received colistin monotherapy or colistin in combination with either sulbactam or carbapenems. It must be emphasized that according to study methodology, sulbactam’s daily dose was 3 g and only two isolates were susceptible to sulbactam.^46^ Moreover, in a prospective cohort study of 182 patients with XDR-*A. baumannii* pneumonia (mainly VAP and to a lesser extent HAP) in Thailand, 92 patients received colistin plus 6 g of sulbactam, whereas 90 patients received colistin in combination with carbapenems. Despite the high sulbactam MIC for isolates and the use of 6 g of this agent per day, overall mortality rates at 7, 14 and 28 days were not significantly different between the two subgroups of patients.^48^ Although controversial, these clinical findings about colistin/sulbactam combination may partially be in accordance with a pharmacokinetics/pharmacodynamics model, which has indicated enhanced bacterial killing of CRAB isolates by the use of both the abovementioned drugs.^49^

Several randomized studies conducted in ICUs in Iran have compared high-dose ampicillin-sulbactam with colistin, each one of these drugs in combination with another agent, for the treatment of MDR-*A. baumannii* VAP. First, Khalili *et al*. studied 47 patients who received meropenem (2 g every 8 hours) combined with either colistin or ampicillin-sulbactam against CRAB-VAP. Clinical improvement of infection was proven in 18/24 and 16/23 of patients in the meropenem/colistin and meropenem/ampicillin-sulbactam group, respectively. Results were also comparable between the two subgroups of patients with respect to microbiological eradication and 28-day mortality rates. Of note, in this trial the total daily dose of administered sulbactam was 6 g.^33^ In another study published in 2019, 28 patients were treated with nebulized colistin in combination with either IV high-dose ampicillin-sulbactam (8 g of sulbactam per day) or IV colistin for the treatment of MDR-*A. baumannii* VAP, with most isolates being sensitive only to colistin. Similar results were indicated regarding clinical efficacy, microbiological response, length of ICU and hospital stay, as well as survival rates among the two treatment arms, whereas a higher incidence of AKI was observed in the IV colistin group of participants.^42^ Finally, in an interim analysis of 23 patients with MDR-*A. baumannii* VAP, participants were randomized to receive colistin plus levofloxacin (*n* = 11) or continuous infusion of ampicillin-sulbactam (24 g daily, i.e. 8 g of sulbactam per day) plus levofloxacin (*n* = 12). Significantly higher clinical response rates (83% versus 27%), as well as significantly lower 28-day (42% versus 82%) and 14-day (8% versus 73%) mortality rates were observed in the ampicillin-sulbactam treatment group. Furthermore, a significantly lower incidence of nephrotoxicity was indicated in the same subgroup of patients.^39^

#### Sulbactam—tigecycline

Limited data exist regarding direct comparison between sulbactam-based and tetracycline-based regimens against CRAB pneumonia. In a multicentre retrospective study including 238 ICU patients with CRAB pneumonia, tigecycline monotherapy and tigecycline-based treatment was compared with sulbactam-based regimens, as well as colistin monotherapy and colistin-combination therapies. The lowest crude ICU mortality (8.3%) was observed in patients treated with sulbactam-based regimens, although only 12 of 238 patients (5%) received this type of treatment, rendering them a small sample size. Overall, tigecycline-based therapy was associated with higher rates of ICU mortality and treatment failure.^36^ Moreover, in a retrospective study conducted in Taiwan, 84 tigecycline-treated were matched to 84 sulbactam-treated individuals (maximum daily dose of sulbactam was 4 g), in combination with other antimicrobials, with respect to the treatment of MDR-*A. baumannii* pneumonia. In the tigecycline group, fewer patients received combination therapy, and more patients had delays in treatment initiation. Although microbiological eradication rate was significantly higher in the sulbactam group, there were no significant differences regarding clinical resolution and mortality rates among the two subgroups of patients.^45^

Some trials have been performed examining combination treatment of tigecycline with cefoperazone-sulbactam. In a randomized controlled trial of 42 patients with XDR-*A. baumannii* VAP, participants received either tigecycline alone or combined with cefoperazone-sulbactam, and combination treatment was associated with higher clinical efficacy rates.^43^ In another study, 114 patients with MDR-*A. baumannii* pulmonary infection were randomized to be treated with either cefoperazone-sulbactam alone or in combination with tigecycline. After 14 days of treatment, inflammatory markers were significantly lower in the combination group of patients.^37^

Of note, the dose of tigecycline used in most of the abovementioned studies was 50 mg twice daily (standard dose). Higher doses (i.e. 200 mg daily) are required for adequate tigecycline concentrations in the ELF and achievement of PK/PD target (*f*AUC/MIC), whereas even these doses may not be appropriate in case of difficult-to-treat pathogens with higher tigecycline MICs.^50^ According to a systematic review and meta-analysis published by Zha *et al*. in 2020, the use of high-dose tigecycline was associated with better clinical outcomes and reduced mortality in the context of severe infections.^51^

#### Comparison of multiple antimicrobial regimens against CRAB pneumonia

Other studies have been carried out, evaluating multiple therapeutic regimens against CRAB pneumonia. In a retrospective cohort study performed in Thailand, survival at 28 days was assessed in 166 patients with XDR-*A. baumannii* pneumonia, who received three different combination treatments. Ninety-three patients were treated with high-dose sulbactam (6 g of sulbactam per day), 43 were treated with 100 mg of tigecycline daily and 30 with a high-dose prolonged infusion of a carbapenem, all these antimicrobials in combination with nebulized or IV colistin. Survival rates at 28 days were not significantly different between the three subgroups of patients. Of note, all isolates were resistant to sulbactam.^34^

Moreover, a systematic review and a Bayesian network meta-analysis (NMA) were performed by Jung *et al*., evaluating 15 different antimicrobial treatments against MDR- and XDR-*A. baumannii* pneumonia.^52^ Twenty-three studies and 2118 adult patients were included in the Bayesian NMA and IV colistin monotherapy was used as a common comparator. Researchers found that sulbactam monotherapy ranked first for reducing all-cause mortality, which was defined as the primary outcome of the study. Four additional therapeutic regimens ranked higher than IV colistin monotherapy, including high-dose sulbactam (i.e. 9 g/day or more), fosfomycin plus IV colistin, inhaled combined with IV colistin and high-dose tigecycline (i.e. 200 mg/day after a loading dose of 200 mg). These regimens also ranked higher than IV colistin monotherapy regarding the effect on clinical cure. Moreover, fosfomycin plus IV colistin ranked highest for microbiological eradication, followed by sulbactam and sulbactam plus inhaled colistin. Overall, sulbactam and inhaled plus IV colistin were found to be significantly superior to IV colistin monotherapy with regards to survival and clinical cure, respectively. Consequently, researchers characterized sulbactam as the most effective treatment to reduce all-cause mortality in critically ill patients with drug-resistant *A. baumannii* pneumonia.^52^

### Sulbactam-based regimens versus other treatments against CRAB bacteraemia

Contrary to pneumonia, evidence from comparative studies between sulbactam-based treatments and other agents against CRAB bacteraemia are limited. A multicentre retrospective study was conducted in 27 tertiary-care centres in Turkey, including 250 patients, who received colistin monotherapy, colistin plus a carbapenem, colistin plus sulbactam (4–6 g per day) or colistin in combination with other antimicrobials for the treatment of XDR-*A. baumannii* bacteraemia. Clinical response, microbiological eradication and 14-day survival rates were significantly higher in the combination than in the colistin-monotherapy group of patients, whereas no statistically significant differences were observed among the three combination subgroups.^29^

Furthermore, in a comparative study of 210 patients with CRAB-bloodstream infection in China, researchers examined the therapeutic effect of tigecycline in contrast to cefoperazone-sulbactam, in combination with other antimicrobial agents. It was indicated that 28-day mortality was significantly lower in the cefoperazone-sulbactam group of patients, and more specifically, mortality seemed to be the lowest when this antimicrobial was combined with imipenem/cilastatin.^40^

### Evaluation of multiple antimicrobial regimens in multiple types of infection

In pursuit of the optimal antibiotic treatment strategy for CRAB infections, larger studies have been conducted, comparing the efficacy of multiple antimicrobial combinations in several types of infection. In a retrospective study of 167 patients with CRAB infections, most of whom had bloodstream infections (47% versus 32% pneumonia and 17% surgical site infections), 82 patients were treated with IV polymyxins and 85 with ampicillin-sulbactam, simultaneously with other antimicrobials. Higher clinical cure and improvement rates, as well as lower mortality rates were observed in the ampicillin-sulbactam-treated group of patients. Multivariate analysis of risk factors associated with mortality indicated that polymyxin therapy, along with higher APACHE II score, septic shock, delays in treatment initiation and renal failure, were independent predictors of mortality until the end of treatment.^41^

In another retrospective cohort study in Taiwan, researchers evaluated 386 patients with healthcare-associated MDR-*A. baumannii* infections, including VAP, HAP, bacteraemia and other sites of infection. One hundred and twenty patients received sulbactam (1 g every 6 hours) plus imipenem/cilastatin, whereas 266 patients received either tigecycline alone or in combination with a carbapenem or piperacillin/tazobactam or a third-generation cephalosporin. Researchers found no significant differences in 30-day mortality and length of hospital and ICU stay between the two subgroups of patients.^35^

Moreover, a retrospective cohort study was carried out in 10 large Korean clinical centres, including 282 ICU adult patients with CRAB infections and evaluating the impact of colistin-, carbapenem- and sulbactam-based regimens and other treatments (i.e. tigecycline-, minocycline- and amikacin-containing regimens) on 7- and 28-day mortality, as well as on clinical and microbiological response.^44^ The most common type of infection was pneumonia (91.1%), followed by primary bacteraemia (7.1%) and urinary tract infection (1.4%). In multivariate analysis, combination treatment of colistin and carbapenem significantly reduced 7-day mortality, sulbactam-containing regimen significantly decreased 28-day mortality, whereas colistin monotherapy significantly increased 28-day mortality. According to subgroup analysis, sulbactam-containing regimen also significantly reduced 28-day mortality in CRAB pneumonia. With respect to clinical improvement, amikacin-containing regimens, sulbactam monotherapy and combination therapy of sulbactam and minocycline were associated with the highest clinical response rates at the end of treatment, although only amikacin-containing regimens exhibited statistically significant results. Moreover, minocycline-containing regimens were associated with the highest microbiological response rates at 14 days, 28 days and at the end of treatment. Of note, all CRAB isolates except one were susceptible to colistin and ∼80% of them to minocycline, whereas the susceptibility rate to ampicillin/sulbactam was <10%. Finally, colistin-containing regimens and tigecyline were associated with increased nephrotoxicity and hepatotoxicity, respectively. Overall, researchers concluded that combination therapy of colistin and carbapenem and sulbactam-based regimens may improve survival rates in CRAB infections.^44^

A series of systematic reviews and meta-analyses have been carried out, assessing the efficacy of sulbactam-based and multiple other regimens in the treatment of CRAB infections. A systematic review and meta-analysis published by Chu *et al*. in 2013, included four studies (three retrospective and one prospective), directly comparing sulbactam-based regimens with other comparator drugs with respect to clinical response rate in CRAB infections.^53^ Two of the studies included patients with CRAB-associated VAP, one study included patients with CRAB-associated bloodstream infections, whereas the researchers in the remaining study did not specify the type of infection. A total of 112 patients received sulbactam in combination with ampicillin, carbapenem or cefoperazone, whereas 107 patients were treated with other regimens, such as colistin, cephalosporins, anti-pseudomonal penicillin agents, fluoroquinolones, minocycline/doxycycline, aminoglycosides, tigecycline, imipenem/cilastatin and combination treatment. According to the results, sulbactam-based therapy did not significantly outperform comparator therapy in terms of the combined clinical response rate odds ratio. However, this study has several limitations because of the small sample size, as well as the heterogeneity in the drugs administered with sulbactam.^53^

In another systematic review, which included 29 studies (4 RCTs and 25 cohort studies) and 2529 patients, researchers thoroughly compared and ranked the efficacy and safety of multiple treatment options in MDR- and XDR-*A. baumannii* infections.^54^ Seventeen studies were carried out on pneumonia (VAP alone or HAP/VAP) and the remaining 12 studies on several types of infection, such as bacteraemia, pneumonia and intra-abdominal infections. According to NMA of 17 studies (1476 patients), there were no statistically significant differences between treatment options regarding clinical cure rate, although the triple combination of colistin, sulbactam and tigecycline had the highest rank among all regimens. As regards microbiological cure rate, the NMA of 20 studies (1863 patients) revealed no significant differences for colistin in combination with other antimicrobials versus colistin in combination with sulbactam, and sulbactam in combination with other agents. Combined treatment of colistin with rifampicin, sulbactam or other antimicrobials, such as carbapenems, exhibited significantly higher microbiological cure rate contrasted to colistin monotherapy. Moreover, tigecycline in monotherapy or in combination with other treatments was found to be less effective than other therapeutic options in terms of microbiological cure rate. Finally, with respect to all-cause mortality as a secondary outcome, colistin combined with other antibiotics was associated with a significantly lower all-cause mortality compared with sulbactam in combination with other agents. Overall, researchers concluded that colistin-combination therapy, especially with sulbactam, indicated an important microbiological benefit, supporting the use of this combination in the treatment of MDR- and XDR-*A. baumannii* infections.^54^

Finally, in a more recent systematic review published by Liu *et al*., researchers compared the efficacy and safety of high-dose sulbactam (i.e. ≥4 g/day) or colistin in combination with other antimicrobials for MDR- and XDR-*A. baumannii* infections.^55^ They included 18 studies (7 RCTs and 11 retrospective studies) and 1835 patients. Eleven studies were performed on pneumonia (VAP alone, HAP alone or VAP/HAP) and seven studies on multiple types of infection, such as pneumonia, as well as bloodstream, urinary tract and post-surgical intra-abdominal infections. Primary outcomes were clinical improvement, clinical cure, microbiological cure and all-cause mortality, whereas a secondary outcome was drug-related nephrotoxicity. The NMAs to evaluate clinical improvement and clinical cure, showed that high-dose sulbactam in combination with another antimicrobial (levofloxacin or tigecycline) were the highest ranking when compared to colistin-based regimens. According to the results of the NMA that assessed the impact of multiple combination therapies on microbiological eradication, there were no statistically significant differences between various colistin-based regimens and high-dose sulbactam in combination with colistin or carbapenems. However, colistin combined with rifampicin, fosfomycin or high-dose sulbactam was significantly superior to colistin monotherapy and high-dose sulbactam in combination with levofloxacin or tigecycline, in terms of microbiological eradication. Moreover, there were no statistically significant differences in all-cause mortality among patients treated with the different antimicrobial agents. As regards the secondary outcome, combination of colistin with high-dose sulbactam or carbapenems was associated with less nephrotoxicity compared with combination of this agent with other antimicrobials. Researchers interpreted their findings, emphasizing that high-dose sulbactam combined with other agents (including colistin) might be a favourable therapeutic option for MDR- and XDR-*A. baumannii* infections.^55^

### The novel β-lactam/β-lactamase inhibitor combination sulbactam/durlobactam

Sulbactam/durlobactam was approved in 2023 by the FDA in the USA for treatment of hospital-acquired bacterial pneumonia and ventilator-associated bacterial pneumonia caused by suspectable ABC complex.^56^ As detailed previously, sulbactam’s activity against ABC can be limited as multiple β-lactamases found in *Acinetobacter* spp. can degrade sulbactam.^11^ Durlobactam’s ability to inhibit a wide range of β-lactamases, including the OXA carbapenemases that are prevalent in MDR-*A. baumannii*, allows for the restoration of sulbactam’s intrinsic activity.^11,23^ Furthermore, due to the improved structure of durlobactam, these enzymes may be bound and inactivated more effectively, resulting in the combination treatment having potent antibacterial activity.^57^

There is a limited number of trials that assessed the role of sulbactam/durlobactam in CRAB infections. The first trial was a multicentre phase 2 trial that compared sulbactam/durlobactam versus placebo in 80 hospitalized patients with complicated urinary tract infections.^58^ Background therapy in both groups consisted of imipenem-cilastatin. Their findings showed that sulbactam/durlobactam was with limited serious side effects, and microbiological success in the modified intent-to-treat participants was similar between both groups (76.6% in the sulbactam/durlobactam group and 81.0% in the placebo group at test-of-care visit). Interestingly, this trial included seven patients with imipenem non-susceptible Gram-negative microbes in which the success rate was 100% for the sulbactam/durlobactam group compared to 75% in the placebo group, however, it is important to note that this was a small and limited sample of patients.^58^ In a separate phase 3 multicentre trial (ATTACK trial), 125 patients with confirmed carbapenem-resistant ABC infections were randomized to receive either colistin or sulbactam–durlobactam, both in combination with imipenem-cilastatin. Compared to the colistin group, patients receiving sulbactam–durlobactam exhibited a lower 28-day all-cause mortality rate (19% versus 32%, CI: −30.0 to 3.5), meeting the trial’s criteria for non-inferiority. The colistin group demonstrated significantly higher number of nephrotoxicity (38% versus 13%, *P* < 0.001) and reported more adverse events leading to antibiotic cessation (16% versus 11%).^32^ This trial formed the basis for the Infectious Diseases Society of America recommendation that sulbactam/durlobactam is the preferred agent, in combination with meropenem or imipenem/cilastatin, for the treatment of CRAB infections.^9^ The rationale behind studying the combination of sulbactam–durlobactam with imipenem could be explained by their synergetic activity against *Acinetobacter* spp. This combination has been shown to effectively inhibits multiple PBPs in *Acinetobacter* spp. Specifically, sulbactam primarily targets PBP1a and PBP3, while imipenem targets PBP1a, PBP2 and PBP3 leading to disruption various stages of cell wall synthesis, thus, enhancing antibacterial efficacy.^59^ Additionally, durlobactam protects both sulbactam and imipenem from degradation by OXA-type carbapenemases by targeting OXA-23 and OXA-51, further contributing to the combination’s effectiveness.^59^ In case that sulbactam/durlobactam is not available, the panel recommends high-dose ampicillin-sulbactam (9 g of sulbactam daily) in combination with at least one other agent, such as polymyxin, minocycline, tigecycline or cefiderocol, as an alternative therapeutic option.^9^

Additionally, several case reports were sought from the literature that demonstrated the role of sulbactam–durlobactam combination therapy in treating CRAB infections. In one case, multiple antibiotic regimens, including polymixins and meropenem, failed to achieve clinical cure in one patient with VAP due to XDR-*A. baumannii*. However, the patient was able to demonstrate rapid and significant improvement once treated with sulbactam–durlobactam and cefiderocol.^60^ A second case reported successful management of necrotizing pneumonia and empyema after the patient received sulbactam–durlobactam combined with meropenem.^61^ In another challenging case, a patient with CRAB meningitis resistant to cefiderocol and high-dose ampicillin-sulbactam was able to achieve clinical recovery and microbiological clearance following administration of sulbactam–durlobactam combined with meropenem.^62^ Another case also discussed the successful use of sulbactam–durlobactam in a patient with CRAB-associated neurosurgical infection and meningitis following emergent left hemicraniectomy requiring ICU admission.^63^ In that case, the patient culture data revealed OXA-producing *A. baumannii* that was first treated with a combination of cefiderocol and polymyxin B and attempts at surgical source controlled, however, with clinical and imaging evidence of worsening infection; therefore, sulbactam–durlobactam was used in combination with cefiderocol and minocycline, which achieved clinical resolution of the infection.^63^

Finally, another case highlighted the role of encoded β-lactamases, such as blaADC-30, blaOXA-23 and blaOXA-66, and mutations in siderophore receptor genes, *piuA* and *fepA*, that contributed to significant resistance and affected iron transport mechanisms carried by cefiderocol. Despite these resistance mechanisms to cefiderocol, sulbactam–durlobactam could achieve complete microbiological clearance of the CRAB infection in a burn patient with VAP, due to durlobactam protecting sulbactam from β-lactamase degradation.^64^ Although these cases provide limited insight, they still highlight the important role sulbactam–durlobactam could play in the treatment of CRAB infections.

### Cefiderocol and sulbactam-based regimens: future perspective for CRAB therapy

A promising combination for infections secondary to CRAB is cefiderocol with sulbactam, albeit, with very limited reports on its utility in clinical practice. However, there are promising data from *in vitro* and *in vivo* studies regarding the role of this combination. An *in vitro* study by Lewis *et al*., assessed the synergistic effect of cefiderocol with other antimicrobial regimens including sulbactam against two strains of CRAB (one strain was cefiderocol resistant and the other was cefiderocol susceptible) using a time–kill curve assay.^65^ The combination of cefiderocol and sulbactam exhibited significant synergistic activity against both cefiderocol-susceptible and cefiderocol-resistant CRAB isolates by showing a significant reduction in bacterial count, indicating a potential role against CRAB infections.^65^ Similar results were also shown in an *in vivo* study by Gill *et al*., using a murine thigh infection model with human-simulated dosing regimens to assess the efficacy of cefiderocol with ampicillin/sulbactam against 15 CRAB isolates.^66^ They showed that this combination resulted in bacterial reduction and prevented the emergence of resistance against cefiderocol when compared to cefiderocol monotherapy.^66^ Finally, a case series by Bavaro *et al*., describes cefiderocol-based combinations in 13 patients for multiresistant Gram-negative infections including CRAB, and three patients received cefiderocol at 2 g every 8 hours, combined with ampicillin/sulbactam at 3 g every 6 hours.^67^ Two of the patients had VAP and one had bacteraemia secondary to CRAB infection with all three achieving microbiological eradication and clinical cure.^67^ These studies highlight the promising potential of combining cefiderocol with sulbactam-based regimens. However, given the limited available data, further studies are warranted to comprehensively assess the safety and efficacy of this combination in larger patient populations.

### Conclusion

Clinical studies that compare sulbactam-based versus other regimens against CRAB infections exhibit diverse results. The main reason is the high heterogeneity between these studies in various parameters, such as sulbactam dosage, sulbactam MICs for *A. baumannii* isolates and antimicrobial combinations used. High-dose sulbactam combined with other agents has demonstrated more encouraging results in some cases, especially against CRAB-associated pneumonia. Its sufficient penetration into pulmonary ELF may render sulbactam a most attractive therapeutic option than other antimicrobials, such as polymyxins, in cases of CRAB-associated VAP or HAP. Sulbactam/durlobactam combined with a carbapenem also constitutes a preferred therapeutic strategy against these difficult-to-treat infections, although clinical data are still limited. More randomized clinical trials comparing sulbactam-based with other regimens are warranted for the purpose of answering several questions regarding the most effective combination, the appropriate dose of the agents used and the number of antimicrobials that should be combined. However, current evidence already suggests that sulbactam may play a significant role in the combination therapy of CRAB infections.

## References

[1] Wong D, Nielsen TB, Bonomo RA et al Clinical and pathophysiological overview of *Acinetobacter* infections: a century of challenges. Clin Microbiol Rev 2017; 30: 409–47. 10.1128/CMR.00058-16 27974412 PMC5217799

[2] Morris FC, Dexter C, Kostoulias X et al The mechanisms of disease caused by *Acinetobacter baumannii*. Front Microbiol 2019; 10: 1601. 10.3389/fmicb.2019.01601 31379771 PMC6650576

[3] Mancuso G, Midiri A, Gerace E et al Bacterial antibiotic resistance: the most critical pathogens. Pathogens 2021; 10: 1310. 10.3390/pathogens10101310 34684258 PMC8541462

[4] Bartal C, Rolston KVI, Nesher L. Carbapenem-resistant *Acinetobacter baumannii*: colonization, infection and current treatment options. Infect Dis Ther 2022; 11: 683–94. 10.1007/s40121-022-00597-w 35175509 PMC8960525

[5] Arulkumaran N, Routledge M, Schlebusch S et al Antimicrobial-associated harm in critical care: a narrative review. Intensive Care Med 2020; 46: 225–35. 10.1007/s00134-020-05929-3 31996961 PMC7046486

[6] Perez F, Hujer AM, Hujer KM et al Global challenge of multidrug-resistant *Acinetobacter baumannii*. Antimicrob Agents Chemother 2007; 51: 3471–84. 10.1128/AAC.01464-06 17646423 PMC2043292

[7] Karakonstantis S, Ioannou P, Samonis G et al Systematic review of antimicrobial combination options for pandrug-resistant *Acinetobacter baumannii*. Antibiotics (Basel) 2021; 10: 1344. 10.3390/antibiotics10111344 34827282 PMC8615225

[8] Shields RK, Paterson DL, Tamma PD. Navigating available treatment options for carbapenem-resistant *Acinetobacter baumannii*-*calcoaceticus* complex infections. Clin Infect Dis 2023; 76: S179–93. 10.1093/cid/ciad094 37125467 PMC10150276

[9] Tamma PD, Heil EL, Justo JA et al Infectious Diseases Society of America 2024 guidance on the treatment of antimicrobial-resistant Gram-negative infections. Clin Infect Dis 2024. 10.1093/cid/ciae403 39108079

[10] Penwell WF, Shapiro AB, Giacobbe RA et al Molecular mechanisms of sulbactam antibacterial activity and resistance determinants in *Acinetobacter baumannii*. Antimicrob Agents Chemother 2015; 59: 1680–9. 10.1128/AAC.04808-14 25561334 PMC4325763

[11] Shapiro AB . Kinetics of sulbactam hydrolysis by β-lactamases, and kinetics of β-lactamase inhibition by sulbactam. Antimicrob Agents Chemother 2017; 61: e01612-17. 10.1128/AAC.01612-17 28971872 PMC5700308

[12] Castanheira M, Mendes RE, Gales AC. Global epidemiology and mechanisms of resistance of *Acinetobacter baumannii*-*calcoaceticus* complex. Clin Infect Dis 2023; 76: S166–78. 10.1093/cid/ciad109 37125466 PMC10150277

[13] Mosqueda N, Gato E, Roca I et al Characterization of plasmids carrying the blaOXA-24/40 carbapenemase gene and the genes encoding the AbkA/AbkB proteins of a toxin/antitoxin system. J Antimicrob Chemother 2014; 69: 2629–33. 10.1093/jac/dku179 24879663

[14] Higgins PG, Dammhayn C, Hackel M et al Global spread of carbapenem-resistant *Acinetobacter baumannii*. J Antimicrob Chemother 2010; 65: 233–8. 10.1093/jac/dkp428 19996144

[15] Müller C, Reuter S, Wille J et al A global view on carbapenem-resistant *Acinetobacter baumannii*. mBio 2023; 14: e0226023. 10.1128/mbio.02260-23 37882512 PMC10746149

[16] Jaruratanasirikul S, Nitchot W, Wongpoowarak W et al Population pharmacokinetics and Monte Carlo simulations of sulbactam to optimize dosage regimens in patients with ventilator-associated pneumonia caused by *Acinetobacter baumannii*. Eur J Pharm Sci 2019; 136: 104940. 10.1016/j.ejps.2019.05.018 31132402

[17] Castanheira M, Mendes RE, Jones RN. Update on *Acinetobacter* species: mechanisms of antimicrobial resistance and contemporary in vitro activity of minocycline and other treatment options. Clin Infect Dis 2014; 59 Suppl 6: S367–73. 10.1093/cid/ciu706 25371512

[18] Jaruratanasirikul S, Wongpoowarak W, Aeinlang N et al Pharmacodynamics modeling to optimize dosage regimens of sulbactam. Antimicrob Agents Chemother 2013; 57: 3441–4. 10.1128/AAC.00342-13 23650160 PMC3697348

[19] Blum RA, Kohli RK, Harrison NJ et al Pharmacokinetics of ampicillin (2.0 grams) and sulbactam (1.0 gram) coadministered to subjects with normal and abnormal renal function and with end-stage renal disease on hemodialysis. Antimicrob Agents Chemother 1989; 33: 1470–6. 10.1128/AAC.33.9.1470 2817847 PMC172685

[20] Reitberg DP, Marble DA, Schultz RW et al Pharmacokinetics of cefoperazone (2.0 g) and sulbactam (1.0 g) coadministered to subjects with normal renal function, patients with decreased renal function, and patients with end-stage renal disease on hemodialysis. Antimicrob Agents Chemother 1988; 32: 503–9. 10.1128/AAC.32.4.503 3377461 PMC172210

[21] Lorenzen JM, Broll M, Kaever V et al Pharmacokinetics of ampicillin/sulbactam in critically Ill patients with acute kidney injury undergoing extended dialysis. Clin J Am Soc Nephrol 2012; 7: 385–90. 10.2215/CJN.05690611 22223613 PMC3302675

[22] Yokoyama Y, Matsumoto K, Ikawa K et al Pharmacokinetics of prophylactic ampicillin–sulbactam and dosing optimization in patients undergoing cardiovascular surgery with cardiopulmonary bypass. Biol Pharm Bull 2015; 38: 1817–21. 10.1248/bpb.b15-00334 26521833

[23] Durand-Réville TF, Guler S, Comita-Prevoir J et al ETX2514 is a broad-spectrum β-lactamase inhibitor for the treatment of drug-resistant Gram-negative bacteria including *Acinetobacter baumannii*. Nat Microbiol 2017; 2: 17104. 10.1038/nmicrobiol.2017.104 28665414

[24] Rodriguez CH, Brune A, Nastro M et al In vitro synergistic activity of the sulbactam/avibactam combination against extensively drug-resistant *Acinetobacter baumannii*. J Med Microbiol 2020; 69: 928–31. 10.1099/jmm.0.001211 32584214

[25] Pasteran F, Cedano J, Baez M et al A new twist: the combination of sulbactam/avibactam enhances sulbactam activity against carbapenem-resistant *Acinetobacter baumannii* (CRAB) isolates. Antibiotics (Basel) 2021; 10: 577. 10.3390/antibiotics10050577 34068158 PMC8152955

[26] Abouelhassan Y, Kuti JL, Nicolau DP et al Pharmacokinetic/pharmacodynamic analysis of sulbactam against *Acinetobacter baumannii* pneumonia: establishing *in vivo* efficacy targets in the epithelial lining fluid. JAC Antimicrob Resist 2024; 6: dlae203. 10.1093/jacamr/dlae203 39712636 PMC11660682

[27] Cheah SE, Wang J, Nguyen VT et al New pharmacokinetic/pharmacodynamic studies of systemically administered colistin against *Pseudomonas aeruginosa* and *Acinetobacter baumannii* in mouse thigh and lung infection models: smaller response in lung infection. J Antimicrob Chemother 2015; 70: 3291–7. 10.1093/jac/dkv267 26318190

[28] Betrosian AP, Frantzeskaki F, Xanthaki A et al High-dose ampicillin-sulbactam as an alternative treatment of late-onset VAP from multidrug-resistant *Acinetobacter baumannii*. Scand J Infect Dis 2007; 39: 38–43. 10.1080/00365540600951184 17366011

[29] Batirel A, Balkan II, Karabay O et al Comparison of colistin-carbapenem, colistin-sulbactam, and colistin plus other antibacterial agents for the treatment of extremely drug-resistant *Acinetobacter baumannii* bloodstream infections. Eur J Clin Microbiol Infect Dis 2014; 33: 1311–22. 10.1007/s10096-014-2070-6 24532009

[30] Betrosian AP, Frantzeskaki F, Xanthaki A et al Efficacy and safety of high-dose ampicillin/sulbactam vs. colistin as monotherapy for the treatment of multidrug resistant *Acinetobacter baumannii* ventilator-associated pneumonia. J Infect 2008; 56: 432–6. 10.1016/j.jinf.2008.04.002 18501431

[31] Kalin G, Alp E, Akin A et al Comparison of colistin and colistin/sulbactam for the treatment of multidrug resistant *Acinetobacter baumannii* ventilator-associated pneumonia. Infection 2014; 42: 37–42. 10.1007/s15010-013-0495-y 23828559

[32] Kaye KS, Shorr AF, Wunderink RG et al Efficacy and safety of sulbactam–durlobactam versus colistin for the treatment of patients with serious infections caused by *Acinetobacter baumannii–calcoaceticus* complex: a multicentre, randomised, active-controlled, phase 3, non-inferiority clinical trial (ATTACK). Lancet Infect Dis 2023; 23: 1072–84. 10.1016/S1473-3099(23)00184-6 37182534

[33] Khalili H, Shojaei L, Mohammadi M et al Meropenem/colistin versus meropenem/ampicillin-sulbactam in the treatment of carbapenem-resistant pneumonia. J Comp Eff Res 2018; 7: 901–11. 10.2217/cer-2018-0037 30192166

[34] Khawcharoenporn T, Pruetpongpun N, Tiamsak P et al Colistin-based treatment for extensively drug-resistant *Acinetobacter baumannii* pneumonia. Int J Antimicrob Agents 2014; 43: 378–82. 10.1016/j.ijantimicag.2014.01.016 24613422

[35] Lee Y-T, Tsao S-M, Hsueh P-R. Clinical outcomes of tigecycline alone or in combination with other antimicrobial agents for the treatment of patients with healthcare-associated multidrug-resistant *Acinetobacter baumannii* infections. Eur J Clin Microbiol Infect Dis 2013; 32: 1211–20. 10.1007/s10096-013-1870-4 23553594

[36] Liang C-A, Lin Y-C, Lu P-L et al Antibiotic strategies and clinical outcomes in critically ill patients with pneumonia caused by carbapenem-resistant *Acinetobacter baumannii*. Clin Microbiol Infect 2018; 24: 908.e1–e7. 10.1016/j.cmi.2017.10.033 29108947

[37] Lv Q, Deng Y, Zhu X et al Effectiveness of cefoperazone-sulbactam alone and combined with tigecycline in the treatment of multi-drug resistant *Acinetobacter baumannii* pulmonary infection. J Coll Physicians Surg Pak 2020; 30: 332–4. 10.29271/jcpsp.2020.03.332 32169149

[38] Makris D, Petinaki E, Tsolaki V et al Colistin versus colistin combined with ampicillin-sulbactam for multiresistant *Acinetobacter baumannii* ventilator-associated pneumonia treatment: an open-label prospective study. Indian J Crit Care Med 2018; 22: 67–77. 10.4103/ijccm.IJCCM_302_17 29531445 PMC5842460

[39] Mosaed R, Haghighi M, Kouchak M et al Interim study: comparison of safety and efficacy of levofloxacin plus colistin regimen with levofloxacin plus high dose ampicillin/sulbactam infusion in treatment of ventilator-associated pneumonia due to multi drug resistant *Acinetobacter*. Iran J Pharm Res 2018; 17: 206–13. https://pmc.ncbi.nlm.nih.gov/articles/PMC6447873/#ref-list1 31011353 PMC6447873

[40] Niu T, Luo Q, Li Y et al Comparison of tigecycline or cefoperazone/sulbactam therapy for bloodstream infection due to carbapenem-resistant *Acinetobacter baumannii*. Antimicrob Resist Infect Control 2019; 8: 52. 10.1186/s13756-019-0502-x 30886705 PMC6404342

[41] Oliveira MS, Prado GVB, Costa SF et al Ampicillin/sulbactam compared with polymyxins for the treatment of infections caused by carbapenem-resistant *Acinetobacter* spp. J Antimicrob Chemother 2008; 61: 1369–75. 10.1093/jac/dkn128 18367459

[42] Pourheidar E, Haghighi M, Kouchek M et al Comparison of intravenous ampicillin-sulbactam plus nebulized colistin with intravenous colistin plus nebulized colistin in treatment of ventilator associated pneumonia caused by multi drug resistant *Acinetobacter baumannii*: randomized open label trial. Iran J Pharm Res 2019; 18: 269–81. 10.22037/ijpr.2019.112466.13775 32802106 PMC7393051

[43] Qin Y, Zhang J, Wu L et al Comparison of the treatment efficacy between tigecycline plus high-dose cefoperazone-sulbactam and tigecycline monotherapy against ventilator-associated pneumonia caused by extensively drug-resistant *Acinetobacter baumannii*. Int J Clin Pharmacol Ther 2018; 56: 120–9. 10.5414/CP203102 29319497

[44] Seok H, Choi WS, Lee S et al What is the optimal antibiotic treatment strategy for carbapenem-resistant *Acinetobacter baumannii* (CRAB)? A multicentre study in Korea. J Glob Antimicrob Resist 2021; 24: 429–39. 10.1016/j.jgar.2021.01.018 33571708

[45] Ye J-J, Lin H-S, Yeh C-F et al Tigecycline-based versus sulbactam-based treatment for pneumonia involving multidrug-resistant *Acinetobacter calcoaceticus-Acinetobacter baumannii* complex. BMC Infect Dis 2016; 16: 374. 10.1186/s12879-016-1717-6 27496018 PMC4975895

[46] Yilmaz GR, Guven T, Guner R et al Colistin alone or combined with sulbactam or carbapenem against *A. baumannii* in ventilator-associated pneumonia. J Infect Dev Ctries 2015; 9: 476–85. 10.3855/jidc.6195 25989167

[47] Zalts R, Neuberger A, Hussein K et al Treatment of carbapenem-resistant *Acinetobacter baumannii* ventilator-associated pneumonia: retrospective comparison between intravenous colistin and intravenous ampicillin-sulbactam. Am J Ther 2016; 23: e78–85. 10.1097/MJT.0b013e3182a32df3 24263165

[48] Ungthammakhun C, Vasikasin V, Changpradub D. Clinical outcomes of colistin in combination with either 6-G sulbactam or carbapenems for the treatment of extensively drug-resistant *Acinetobacter baumannii* pneumonia with high MIC to sulbactam, a prospective cohort study. Infect Drug Resist 2019; 12: 2899–904. 10.2147/IDR.S225518 31571943 PMC6750850

[49] Bian X, Liu X, Feng M et al Enhanced bacterial killing with colistin/sulbactam combination against carbapenem-resistant *Acinetobacter baumannii*. Int J Antimicrob Agents 2021; 57: 106271. 10.1016/j.ijantimicag.2020.106271 33352235

[50] De Pascale G, Lisi L, Ciotti GMP et al Pharmacokinetics of high-dose tigecycline in critically ill patients with severe infections. Ann Intensive Care 2020; 10: 94. 10.1186/s13613-020-00715-2 32661791 PMC7357259

[51] Zha L, Pan L, Guo J et al Effectiveness and safety of high dose tigecycline for the treatment of severe infections: a systematic review and meta-analysis. Adv Ther 2020; 37: 1049–64. 10.1007/s12325-020-01235-y 32006240 PMC7223407

[52] Jung SY, Lee SH, Lee SY et al Antimicrobials for the treatment of drug-resistant *Acinetobacter baumannii* pneumonia in critically ill patients: a systemic review and Bayesian network meta-analysis. Crit Care 2017; 21: 319. 10.1186/s13054-017-1916-6 29262831 PMC5738897

[53] Chu H, Zhao L, Wang M et al Sulbactam-based therapy for *Acinetobacter baumannii* infection: a systematic review and meta-analysis. Braz J Infect Dis 2013; 17: 389–94. 10.1016/j.bjid.2012.10.029 23602463 PMC9428054

[54] Kengkla K, Kongpakwattana K, Saokaew S et al Comparative efficacy and safety of treatment options for MDR and XDR *Acinetobacter baumannii* infections: a systematic review and network meta-analysis. J Antimicrob Chemother 2018; 73: 22–32. 10.1093/jac/dkx368 29069421

[55] Liu J, Shu Y, Zhu F et al Comparative efficacy and safety of combination therapy with high-dose sulbactam or colistin with additional antibacterial agents for multiple drug-resistant and extensively drug-resistant *Acinetobacter baumannii* infections: a systematic review and network meta-analysis. J Glob Antimicrob Resist 2021; 24: 136–47. 10.1016/j.jgar.2020.08.021 32889142

[56] Keam SJ . Sulbactam/durlobactam: first approval. Drugs 2023; 83: 1245–52. 10.1007/s40265-023-01920-6 37523122

[57] El-Ghali A, Kunz Coyne AJ, Caniff K et al Sulbactam-durlobactam: a novel β-lactam-β-lactamase inhibitor combination targeting carbapenem-resistant *Acinetobacter baumannii* infections. Pharmacotherapy 2023; 43: 502–13. 10.1002/phar.2802 37052117

[58] Sagan O, Yakubsevitch R, Yanev K et al Pharmacokinetics and tolerability of intravenous sulbactam-durlobactam with imipenem-cilastatin in hospitalized adults with complicated urinary tract infections, including acute pyelonephritis. Antimicrob Agents Chemother 2020; 64: e01506-19. 10.1128/AAC.01506-19 PMC7038258 31843995

[59] Veeraraghavan B, Shin E, Bakthavatchalam YD et al A microbiological and structural analysis of the interplay between sulbactam/durlobactam and imipenem against penicillin-binding proteins (PBPs) of *Acinetobacter* spp. Antimicrob Agents Chemother 2025; 69: e01627-24. 10.1128/aac.01627-24 40035550 PMC11963609

[60] Zaidan N, Hornak JP, Reynoso D. Extensively drug-resistant *Acinetobacter baumannii* nosocomial pneumonia successfully treated with a novel antibiotic combination. Antimicrob Agents Chemother 2021; 65: e0092421. 10.1128/AAC.00924-21 34370576 PMC8522738

[61] Holger DJ, Kunz Coyne AJ, Zhao JJ et al Novel combination therapy for extensively drug-resistant *Acinetobacter baumannii* necrotizing pneumonia complicated by empyema: a case report. Open Forum Infect Dis 2022; 9: ofac092. 10.1093/ofid/ofac092 35350174 PMC8946682

[62] Tamma PD, Immel S, Karaba SM et al Successful treatment of carbapenem-resistant *Acinetobacter baumannii* meningitis with sulbactam-durlobactam. Clin Infect Dis 2024; 79: 819–25. 10.1093/cid/ciae210 38630890 PMC11478584

[63] Snowdin JW, Mercuro NJ, Madaio MP et al Case report: successful treatment of OXA-23 *Acinetobacter baumannii* neurosurgical infection and meningitis with sulbactam-durlobactam combination therapy. Front Med 2024; 11: 1381123. 10.3389/fmed.2024.1381123 PMC11135601 38813376

[64] Tiseo G, Giordano C, Leonildi A et al Salvage therapy with sulbactam/durlobactam against cefiderocol-resistant *Acinetobacter baumannii* in a critically ill burn patient: clinical challenges and molecular characterization. JAC Antimicrob Resist 2023; 5: dlad078. 10.1093/jacamr/dlad078 37325251 PMC10265591

[65] Lewis RE, Palombo M, Diani E et al Synergistic activity of cefiderocol in combination with avibactam, sulbactam or tazobactam against carbapenem-resistant Gram-negative bacteria. Cells 2024; 13: 1315. 10.3390/cells13161315 39195205 PMC11352988

[66] Gill CM, Santini D, Takemura M et al In vivo efficacy & resistance prevention of cefiderocol in combination with ceftazidime/avibactam, ampicillin/sulbactam or meropenem using human-simulated regimens versus *Acinetobacter baumannii*. J Antimicrob Chemother 2023; 78: 983–90. 10.1093/jac/dkad032 36775993 PMC10068413

[67] Bavaro DF, Belati A, Diella L et al Cefiderocol-based combination therapy for “difficult-to-treat” Gram-negative severe infections: real-life case series and future perspectives. Antibiotics (Basel) 2021; 10: 652. 10.3390/antibiotics10060652 34072342 PMC8227820

